# Anemoside A_3_ ameliorates experimental autoimmune encephalomyelitis by modulating T helper 17 cell response

**DOI:** 10.1371/journal.pone.0182069

**Published:** 2017-07-31

**Authors:** Fanny C. F. Ip, Yu Pong Ng, Terry C. T. Or, Peiran Sun, Guangmiao Fu, Jessica Y. H. Li, Wen-Cai Ye, Tom H. Cheung, Nancy Y. Ip

**Affiliations:** 1 Division of Life Science, The Hong Kong University of Science and Technology, Clear Water Bay, Hong Kong, China; 2 Molecular Neuroscience Center, The Hong Kong University of Science and Technology, Clear Water Bay, Hong Kong, China; 3 State Key Laboratory of Molecular Neuroscience, The Hong Kong University of Science and Technology, Clear Water Bay, Hong Kong, China; 4 Guangdong Provincial Key Laboratory of Brain Science, Disease and Drug Development, HKUST Shenzhen Research Institute, Shenzhen, Guangdong, China; 5 HKUST–Jinan Joint Laboratory of Innovative Drug Discovery, Jinan University, Guangzhou, China; 6 Institute of Traditional Chinese Medicine and Natural Products College of Pharmacy, Jinan University, Guangzhou, China; 7 Guangdong Province Key Laboratory of Pharmacodynamic Constituents of Traditional Chinese Medicine and New Drugs Research, Jinan University, Guangzhou, China; Wayne State University, UNITED STATES

## Abstract

Anemoside A_3_ (AA3) is a natural triterpenoid glycoside isolated from the root of *Pulsatilla chinensis* (Bunge) Regel. We previously showed that AA3 exhibits cognitive-enhancing and neuroprotective properties. In the present study, we demonstrated that AA3 modulates inflammatory responses by regulating prostaglandin E receptor 4 signaling. Because prostaglandin E receptor 4 is involved in the pathophysiology of experimental autoimmune encephalomyelitis (EAE), an animal model of human multiple sclerosis (MS), we assessed the beneficial effect of AA3 in EAE mice. AA3 treatment significantly reduced clinical severity and inflammatory infiltrates in the spinal cord of EAE mice. *In vitro* studies revealed that AA3 inhibited the T cell response toward the encephalitogenic epitope of myelin oligodendrocyte glycoprotein (MOG). AA3 significantly downregulated the expressions of certain Th1 and Th17 cytokines in activated T cells re-stimulated by MOG. Moreover, AA3 inhibited the activation of STAT4 and STAT3, which are the transcription factors pivotal for Th1 and Th17 lineage differentiation, respectively, in activated T cells. Pharmacological analysis further suggested that AA3 reduced Th17 cell differentiation and expansion. In conclusion, AA3 exerts an immunomodulatory effect in EAE, demonstrating its potential as a therapeutic agent for MS in humans.

## Introduction

Multiple sclerosis (MS) is a chronic neurological disease with characteristic pathological findings of episodic neurologic dysfunction, perivascular inflammation, demyelination, and axon degeneration in the central nervous system (CNS). Although its etiology remains unknown, strong evidence suggests the involvement of autoimmune mechanisms [1–3]. Patients with MS have many autoreactive T cells against neuro-antigens such as myelin basic protein and myelin oligodendrocyte glycoprotein (MOG). Histological investigation of the white matter from patients with MS shows that the demyelinating plaques are distributed within the optic nerves, brainstem, cerebellum, and spinal cord [4]. These demyelinating plaques can subsequently lead to axonal damage and contribute to disability [2, 3, 5].

Autoreactive type-1 T helper (Th1) and Th17 cells are crucial in the pathogenesis of MS [6–9]. Thus, regulating of T helper cell responses is a promising approach for the treatment of MS. Mounting evidence indicates that prostaglandin E2 (PGE2) signaling profoundly influences the pattern of CD4^+^ T cell responses in neuroinflammatory disorders including MS [7, 10, 11]. PGE2 is a prostaglandin, which are biologically active compounds found virtually in all tissues and organs. Prostaglandins play major roles in the mediation and modulation of pain and inflammation, and are targets of non-steroidal anti-inflammatory drugs (NSAIDs). PGE2 exerts its effects through four G-protein-coupled receptor subtypes that trigger diverse functional responses: EP1, EP2, EP3, and EP4 [12, 13]. Emerging evidence suggests that PGE2 is associated with the pathogeneses of MS in an animal model that exhibits MS-like pathology, termed “experimental autoimmune encephalomyelitis” (EAE) [7, 10, 11, 13]. Prostaglandin levels are elevated in the spinal cord in EAE mice and in the cerebrospinal fluid of patients with MS [10, 11]. Furthermore, the expressions of EP1, EP2, and EP4 are elevated in EAE lesions [10]. In PGE2 receptor-deficient mice, only EP4-knockout mice exhibit significant suppression of EAE [7, 11]. Thus, PGE2–EP4 signaling is a promising target for the development of therapeutics for MS.

The dried root extracts of *Pulsatilla chinensis* (Bunge) Regel (*bai tou weng* in Chinese) have been used in traditional Chinese medicine to treat amoebic dysentery, malaria, bacterial infections, vaginal trichomoniasis, and malignant tumors [14, 15]. Chemical and pharmacological studies demonstrate that triterpenoid glycosides are important bioactive constituents in this plant [14, 16–18]. Among them, anemoside A_3_ (AA3), a natural triterpenoid glycoside isolated from the root of *P*. *chinensis* [14, 17, 18], induces relaxation in rat renal arteries through the stimulated release of endothelium-derived hyperpolarizing factor, activation of K^+^ channel, and inhibition of Ca^2+^ influx [19]. AA3 protects PC12 cells from cell death induced by sodium cyanide or glucose deprivation [20]. Furthermore, we previously demonstrated that AA3 enhances cognition in mice via the regulation of synaptic functions and its neuroprotective effect [21].

Accordingly, in the present study, we showed that AA3 inhibits the activation of PGE2–EP4 signaling and reduces inflammatory injury in EAE mice. Moreover, AA3 improves the clinical scores and reduces spinal cord inflammation in EAE mice. Mechanistically, AA3 attenuates the MOG-induced differentiation of Th1 and Th17 cells, and their signature cytokines in EAE mice during disease progression. In addition, AA3 reduces Th17 differentiation and expansion driven by pro-inflammatory cytokines and PGE2. Our findings collectively demonstrate that AA3 incurs beneficial effects in EAE, suggesting its therapeutic potential for MS treatment.

## Materials and methods

### Preparation of AA3

We purified AA3 (C_41_H_66_O_12_, molecular weight: 750.98) from *P*. *chinensis* roots as described previously [14]. We identified the molecular structure of AA3 by proton nuclear magnetic resonance, carbon-13 nuclear magnetic resonance, and mass spectroscopy. Nuclear magnetic resonance indicated that the purity of AA3 was >95%. We prepared stock solution in 100% DMSO (Sigma-Aldrich, St. Louis, MO, USA) and stored it at −80°C. The final concentration of DMSO in the assay was <0.5%.

### Cell cultures

We maintained human THP-1 acute monocytic leukemia cells in RPMI 1640 medium (Invitrogen, Carlsbad, CA, USA) supplemented with 2 mM l-glutamine, 1 mM sodium pyruvate, 0.05 mM 2-merceptoethanol, 100 units/mL penicillin and streptomycin, and 10% (v/v) heat-inactivated fetal bovine serum (FBS) at 37°C in 5% CO_2_. For experiments, we seeded cells (2 × 10^5^ /mL) in the same medium.

We isolated primary lymphocytes from mouse spleens using Lympholyte-M according to the manufacturer’s protocol (Cedarlane Laboratories, Hornby, Canada). We sieved mouse spleens from 12-week-old C57BL/6 mice through a 70-μm cell strainer (Falcon, Becton Dickinson, Franklin Lakes, NJ, USA), resuspended them in RPMI 1640 medium, and layered them on top of an equal volume of Lympholyte-M. We established the gradient by centrifugation at 1500 × *g* for 20 min to absorb the mononuclear cell layer. We subsequently washed the cells twice with RPMI 1640 medium and subsequently resuspended them in RPMI 1640 containing 10% (v/v) heat-inactivated FBS, and 100 units/mL penicillin and streptomycin. We evaluated cell survival by Trypan Blue staining (Sigma-Aldrich).

We isolated CD4^+^ T cells from spleen lymphocytes by using a CD4^+^ T cell isolation kit II according to the manufacturer’s protocol (Miltenyi Biotech Ltd., Surrey, UK). We retained the magnetically labeled non-target cells in the column in the magnetic separator while the unlabeled cells passed through the column. Then, we collected the isolated CD4+ T cells and resuspended them in RPMI 1640 medium containing 10% (v/v) heat-inactivated FBS, and 100 units/mL penicillin and streptomycin. We determined cell viability and number by Trypan Blue staining.

### Cell proliferation and viability assays

We performed an MTT (3-[4,5-dimethylthiazol-2-yl]-2,5-diphenyltetrazolium bromide) assay to measure the cell viability of the THP-1 cells and primary lymphocytes after 2 days of treatment (USB, Cleveland, OH, USA). To examine the cytotoxic effect of AA3 on naïve CD4+ T cells, we determined cell viability and number by Trypan Blue staining after 4 days of treatment.

### cAMP assay

We pretreated THP-1 cells with 3-isobutyl-1-methylxanthine (1 mM) for 30 min, followed by PGE2 or PGE1-OH (an EP4 agonist; Cayman Chemical, Ann Arbor, MI, USA) stimulation for another 30 min in the presence of L-161982 (an EP4 antagonist; Cayman Chemical) or AA3. We subsequently determined cAMP levels in THP-1 cells using the cAMP-Screen Immunoassay System (Life Technologies, Grand Island, NY, USA).

### Induction of EAE and AA3 treatment

We obtained female, 10-week-old, C57BL/6 mice from The Hong Kong University of Science and Technology Animal Care Facility and acclimated them at the laboratory for one week before immunization. All animal experimental procedures were approved by the Hong Kong University of Science and Technology Animal Ethics Committee (Permit Number: 2013030) and conducted in accordance with the Code of Practice for Care and Use of Animals for Experimental Purposes (Animal Welfare Advisory Group, Agriculture, Fisheries and Conservation Department, Hong Kong). All surgery was performed under isoflurane anesthesia and all efforts were made to minimize suffering.

We randomly assigned C57BL/6 mice to receive EAE induction and/or AA3 treatment. For EAE induction, we subjected the mice to anesthesia with 1% isoflurane (Patterson Veterinary, Devens, MA, USA) for 5 min, and then immunized them with 100 μg MOG_35–55_ peptide in complete Freund’s adjuvant (CFA) containing 2 mg/mL heat-killed *Mycobacterium tuberculosis* according to the manufacturers’ protocol (Hooke Laboratories, Lawrence, MA, USA). The control group was only injected with CFA. We immediately intraperitoneally injected the mice with 0.5 mg/mL pertussis toxin (400 ng). We administered an additional dose of pertussis toxin after 24 h of immunization.

We fed the mice AA3 (100 mg/kg, 10 mL/kg) prepared as a homogeneous suspension in water or water alone by oral gavage on the day after immunization (day 0) or day 8. We continued treatment daily throughout the experiment.

We conducted all animal assessments in a blinded manner. We recorded body weight and clinical signs of disease severity daily. We scored clinical signs of EAE as follows: 0, no clinical signs; 0.5, partially paralyzed tail; 1, paralyzed tail; 2, hindlimb paresis; 2.5, one hindlimb paralyzed; 3, both hindlimbs paralyzed; 3.5, hindlimbs paralyzed and weakness in forelimbs; 4, forelimbs paralyzed; 5, moribund [22]. If the EAE score exceeded 3.5, we sacrificed the animal by decapitation.

### Histological analysis

On day 20 post-immunization, mice were given choral hydrate (Sigma-Aldrich) administration followed by transcardial perfusion with 4% paraformaldehyde. We resected the spinal cords and postfixed them overnight. We stained paraffin-embedded spinal cord sections (10 μm) with hematoxylin and eosin (Bio-Rad Laboratories, Hercules, CA, USA) to assess inflammation and Luxol Fast Blue—Cresyl Echt Violet (American MasterTech Scientific, Inc., Lodi, CA, USA) to assess demyelination, according to the manufacturers’ instructions.

### *Ex vivo* and *in vitro* studies of MOG-specific lymphocytes

We sacrificed the control EAE and AA3-treated EAE mice by decapitation to collect spleens on day 20 post-immunization, subsequently pooling the spleens from individual treatment groups. We isolated lymphocytes using Lympholyte-M (Cedarlane Laboratories) as described previously and cultured them in RPMI 1640 medium containing 10% (v/v) heat-inactivated FBS, and 100 units/mL penicillin and streptomycin. We incubated cultures at 37°C in 5% CO_2_ in the presence or absence of MOG_35–55_ peptide (25 μg/mL) and AA3 (30 μM) for 48 h.

### Quantification of cytokine production

We treated lymphocytes isolated from the experimental mice with the MOG_35–55_ peptide (25 μg/mL) and AA3 (30 μM) for 48 h. We measured the concentrations of interferon (IFN)-γ, interleukin (IL)-4, and IL-17 by ELISA (R&D Systems, Inc., Minneapolis, MN, USA). For Th17 cell expansion, we stimulated CD4^+^ T cells isolated from normal mice with plate-bound anti-CD3ε (5 μg/mL; eBioscience, Frankfurt, Germany) and anti-CD28 (1 μg/mL; eBioscience) in the presence of IL-23 (10 ng/mL; R&D Systems) and AA3 (30 μM) for 4 days [7]. We collected culture media for IL-17 ELISA (R&D Systems).

### Western blot analysis

We cultured lymphocytes from the control EAE mice in the presence or absence of MOG_35–55_ peptide (25 μg/mL) and AA3 (3 and 30 μM) for 48 h. We lysed cells with RIPA lysis buffer with 150 mM NaCl, 1% Nonidet P-40, 1 mM EDTA, 0.5% deoxycholic acid, 2 μg/mL aprotinin, 1 mM PMSF, 5 mM benzamidine, 1 mM sodium orthovanadate, and 10 μg/mL soybean trypsin inhibitor in 50 mM Tris buffer (pH 7.4). We purchased protein quantification reagent from Bio-Rad. Following separation by SDS-PAGE, we transferred the proteins onto a nitrocellulose membrane. After blocking with 0.1% Tween-20 and 5% non-fat dry milk in Tris-buffered saline at room temperature for 1 h, we incubated the membrane with primary antibody (1:1000) overnight at 4°C with horseradish peroxidase (HRP)-conjugated secondary antibody (1:2000) for 1 h. We purchased antibodies against phospho-signal transducer and activator of transcription (STAT) 3, phospho-STAT6, STAT3, STAT4, and STAT6 as well as secondary antibodies (i.e., HRP-conjugated goat anti-mouse or anti-rabbit antibodies) from Cell Signaling Technology (Beverly, MA, USA). Antibodies against phospho-STAT4 and α-tubulin were from BD Biosciences (Lincoln Park, NJ, USA) and Sigma-Aldrich, respectively. We performed detection using the Enhanced Chemiluminescence Western Blot System (GE Healthcare, Buckinghamshire, UK).

### Reverse transcription and real time polymerase chain reaction

We extracted total RNA using the RNeasy Mini Kit (Qiagen, Hilden, Germany). We reverse-transcribed 5 μg total RNA of each sample using oligo (dT) primers and SuperScript II reverse transcriptase (Invitrogen) in a 20-μL volume. We quantified the target genes with a Power SYBR Green PCR master mix kit using a 7500 Fast-real time PCR system according to the manufacturer’s instructions (Applied Biosystems, Foster City, CA, USA). We confirmed the specificity of the SYBR Green PCR signal by melting curve analysis. We used the following primer sequences: forkhead box P3 (*foxp3)* forward primer, 5′-CCTGCCTTGGTACATTCGTG-3′; *foxp3* reverse primer, 5′-TGTTGTGGGTGAGTGCTTTG-3′ [23]; *il-17* forward primer, 5′-TTTAACTCCCTTGGCGCAAAA-3′; *il-17* reverse primer, 5′-CTTTCCCTCCGCATTGACAC-3′ [24]. In each experiment, we used mouse glyceraldehyde-3-phosphate dehydrogenase (*gapdh*) mRNA as an endogenous reference with the following primer sequences: forward primer, 5′-TGCACCACCAACTGCTTAGC-3′; reverse primer, 5′-GGCATGGACTGTGGTCATGAG-3′.

### Flow cytometric analysis of CD4^+^ Th17 cells

For the Th17 cell re-stimulation experiment [7], we first stimulated CD4^+^ T cells for 3 days with plate-bound anti-CD3ε (10 μg/mL), anti-CD28 (10 μg/mL), TGF-β1 (1 ng/mL), and IL-6 (50 ng/mL) in RPMI 1640 medium containing 10% (v/v) heat-inactivated FBS, and 100 units/mL penicillin and streptomycin. We washed cells twice in phosphate-buffered saline, and then re-stimulated them with plate-bound anti-CD3ε (10 μg/mL), anti-CD28 (10 μg/mL), IL-23 (10 ng/mL), and PGE2 (100 nM; Cayman Chemical) in RPMI 1640 medium containing 10% (v/v) heat-inactivated FBS, and 100 units/mL penicillin and streptomycin for another 3 days. For the unstimulated control, we first stimulated CD4^+^ T cells for 3 days with plate-bound anti-CD3ε (10 μg/mL) and anti-CD28 (10 μg/mL) in RPMI 1640 medium containing 10% (v/v) heat-inactivated FBS, and 100 units/mL penicillin and streptomycin without the addition of other cytokines. We washed cells twice in phosphate-buffered saline, and then cultured them with plate-bound anti-CD3ε (10 μg/mL) and anti-CD28 (10 μg/mL) in RPMI 1640 medium containing 10% (v/v) heat-inactivated FBS, and 100 units/mL penicillin and streptomycin for another 3 days. To study the effect of AA3 on Th17 cell differentiation, AA3 (30 μM) was present in the culture medium throughout the 6-day culture period.

On the day of flow cytometric analysis, we treated the cultures with phorbol 12-myristate 13-acetate (50 ng/mL; Sigma-Aldrich) and ionomycin (500 ng/mL; Sigma-Aldrich) together with GolgiPlug (BD Biosciences) for 6 h. We permeabilized and fixed cells in Cytofix/Cytoperm solutions (BD Biosciences) for 15 min at room temperature. After washing, we performed intracellular staining with FITC-conjugated antibody against IFN-γ (BD Biosciences) and PE-conjugated antibody against IL-17A (BD Biosciences) as well as surface antigen staining for CD4 with PerCP-Cy5.5-conjugated antibody (BD Biosciences). We quantified stained cells by using a FACS Aria flow cytometer and analyzed the data using FACSDiva version 6.1.3 (BD Biosciences).

### Statistical analysis

All results are expressed as mean ± SEM. We analyzed data by two-tailed Student’s *t*-tests or one-way ANOVA using Prism software v5 (GraphPad, La Jolla, CA, USA). The level of significance was set at *p* < 0.05.

## Results

### AA3 modulates the PGE2 and EP4 signaling pathways

**Fig 1:**
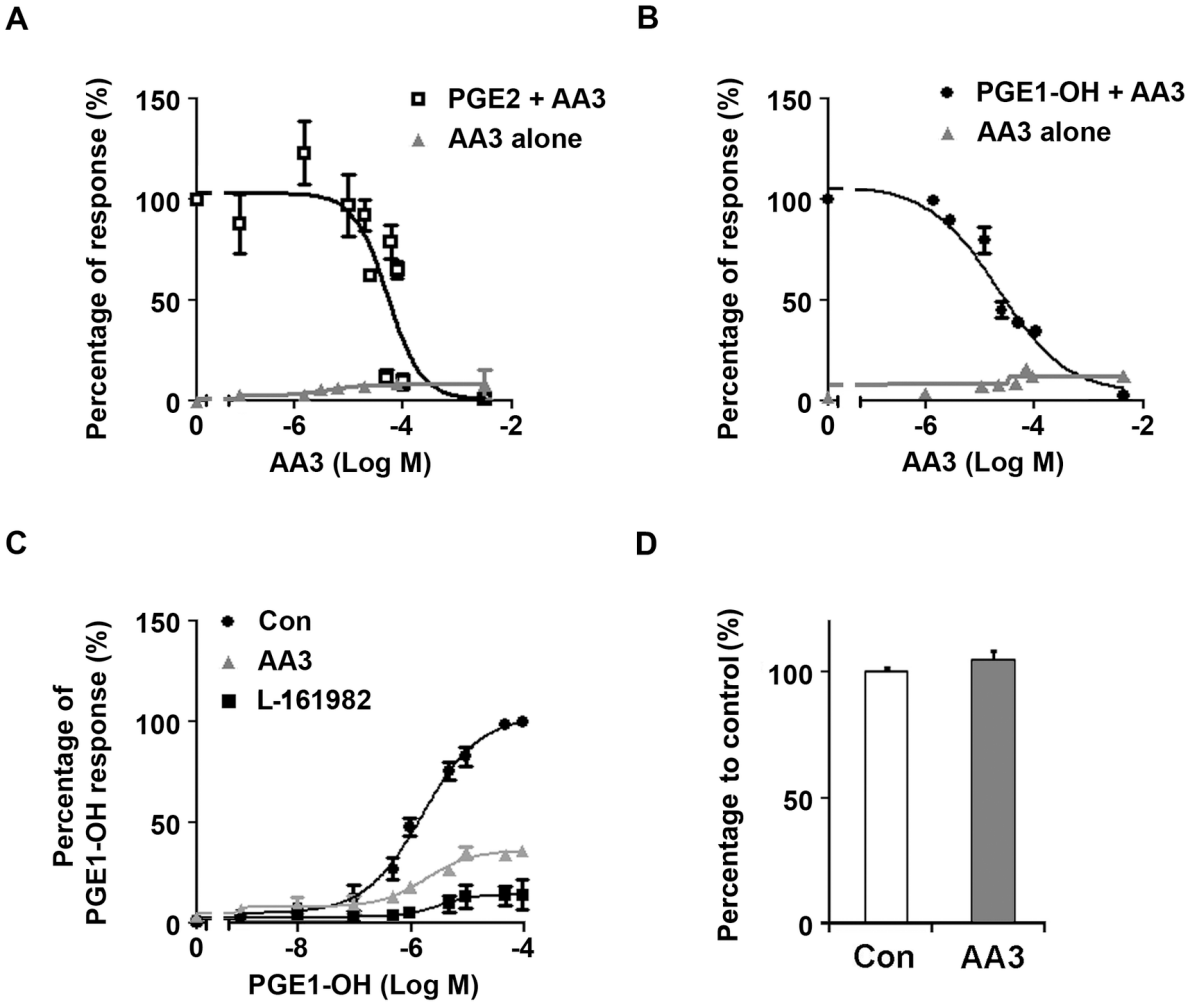
AA3 inhibits PGE2–EP4 signaling. We treated THP-1 cells with PGE2 or PGE-1-OH (an EP4 agonist) in the presence of increasing concentrations of AA3 (A and B, respectively) or with increasing concentrations of PGE-1-OH together with AA3 or L-161982 (C). We measured cellular cAMP levels, and the concentration—response curves are shown. (D) We examined THP-1 cell survival after 2 days of AA3 treatment by MTT assay. Data are mean ± SEM (*n* = 3 per treatment group).

We first examined whether AA3 regulates PGE2–EP4 signaling. PGE2–EP4 activation increases the Gs-mediated activation of adenylate cyclase and the subsequent increase in intracellular cAMP [12, 13]. Following stimulation with PGE2, the level of cAMP increased in THP-1 cells, which was inhibited by AA3 with a half maximal inhibitory concentration (IC_50_) of 22.4 ± 5.8 μM (Fig 1A). AA3 alone had a negligible effect on cAMP level. We then assessed the effect of AA3 on EP4-mediated cAMP response. Treatment with an EP4 selective agonist, PGE1-OH, increased the intracellular level of cAMP; this effect was suppressed by AA3 with an IC_50_ of 22.9 ± 8.1 μM (Fig 1B). To confirm that AA3 modulates EP4 signaling, we treated THP-1 cells with increasing concentrations of PGE1-OH alone or in the presence of L-161982 or AA3 (Fig 1C). The level of cAMP increased by PGE1-OH treatment was blocked by the EP4 inhibitor, L-161982. This finding indicates that PGE1-OH induces cAMP acting through EP4 receptor and that AA3 inhibits EP4-mediated cAMP production. Meanwhile, AA3 treatment did not affect the survival of THP-1 cells, indicating that the reduction of PGE2 and PGE1-OH responses by AA3 is not due to cell death (Fig 1D).

### AA3 attenuates disease severity in EAE mice

**Fig 2:**
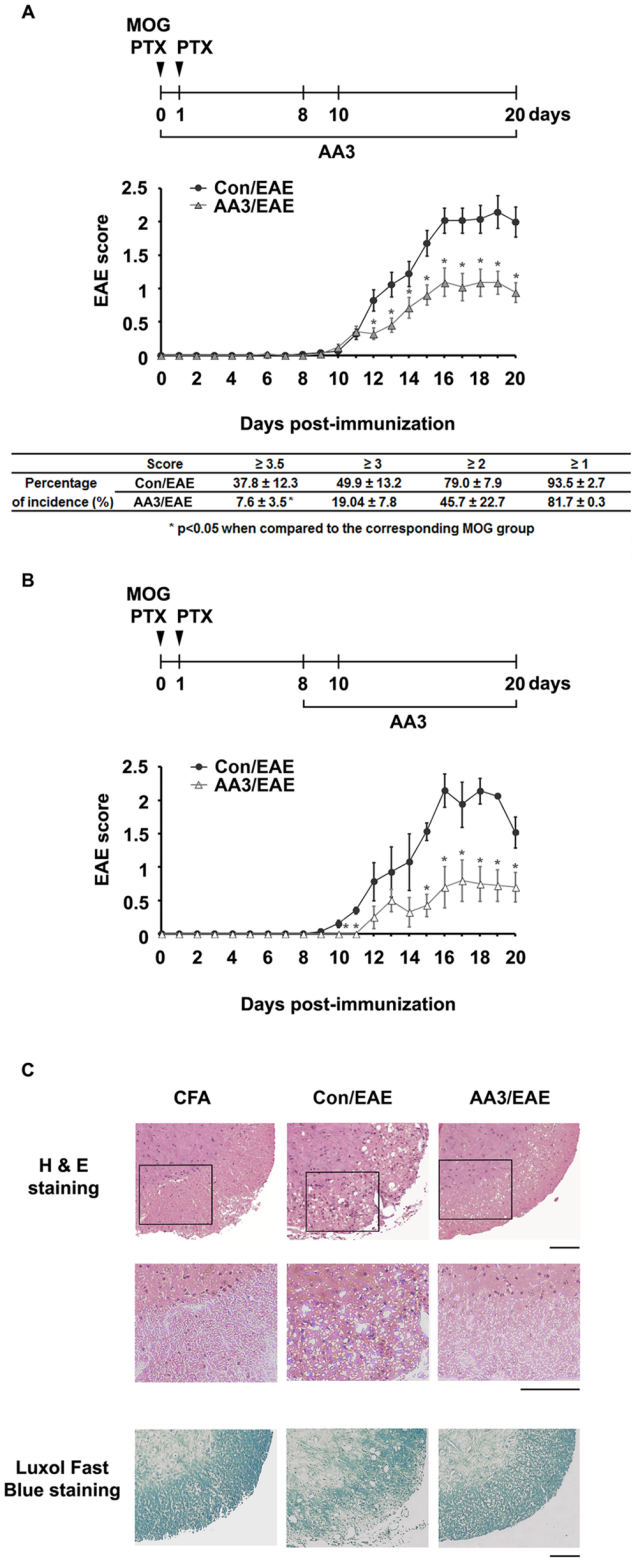
AA3 reduces the disease severity of EAE mice. (A) We orally administered AA3 or water (control) daily starting from the day of immunization. The time courses of symptom development (reflected by the EAE score) and the incidence of EAE scores at day 20 are shown (table below graph). (B) We orally administered AA3 or water (control) to EAE mice daily starting from day 8 post-immunization; the time courses of EAE scores are shown. Data are mean ± SEM (*n* = 24 per treatment group); *p < 0.05, Student’s *t*-test. (C) Histopathologic examination of spinal cord tissues by hematoxylin and eosin (top and middle panels) and Luxol Fast Blue staining (bottom panels). We obtained tissues from mice injected with complete Freund’s adjuvant (CFA), or EAE mice treated with water (Con/EAE) or AA3 (AA3/EAE). Representative pictures are shown. Scale bar, 100 μm.

To evaluate the effect of AA3 in EAE, we immunized C57BL/6 mice with MOG_35–55_ and orally administered AA3 or water daily until the end of the experiment. The first clinical sign, i.e., partial tail paralysis, was observed on day 11 and became serious over the next 6–7 days (Fig 2A). AA3 administration reduced the disease symptoms in the EAE mice (Fig 2A). The mean time for the clinical score to reach tail paralysis (score: 1) in the vehicle control- mice was 13.1 ± 0.5 days, and that in the AA3-treated EAE mice was 15.6 ± 0.1 days (*p* = 0.003, Student’s *t*-test). Furthermore, AA3 treatment reduced the weight loss of the EAE mice. The percentage of weight loss was significantly higher in the control EAE mice than the AA3-treated EAE mice on day 20 post-immunization (14.6 ± 1.1% vs. 9.5 ± 0.1%, respectively, *p* = 0.002, Student’s *t*-test). AA3 not only affected the pre-symptomatic phase of EAE, but also the symptomatic phase. The EAE mice that received delayed AA3 treatment, starting daily on day 8 post-immunization, also exhibited reduced clinical scores (Fig 2B). We did not observe any animal death in any treatment group.

Spinal cord inflammation and demyelination occur in EAE mice during the advanced disease stage [25, 26]. To determine the effect of AA3 on CNS inflammation and demyelination, we performed histological analysis of lumbar spinal cord sections of the EAE mice treated with vehicle control or AA3. The spinal cords of the control EAE mice exhibited substantial inflammation as indicated by vacuolization (i.e., spongiosis) of the white matter and leukocyte infiltration (Fig 2C, top and middle panels). Meanwhile, vacuolization and infiltration of inflammatory cells were reduced in the AA3-treated EAE mice. Demyelinating lesions were more evident in the control EAE mice (Fig 2C, bottom panels), whereas fewer lesions were observed in the AA3-treated EAE mice. Thus, the typical histological characteristics of EAE, including vacuolization, infiltration of inflammatory cells, and regions of demyelination within the spinal cord, were reduced in the AA3-treated EAE mice.

### AA3 modulates Th1, Th2, and Th17 responses in EAE mice

**Fig 3:**
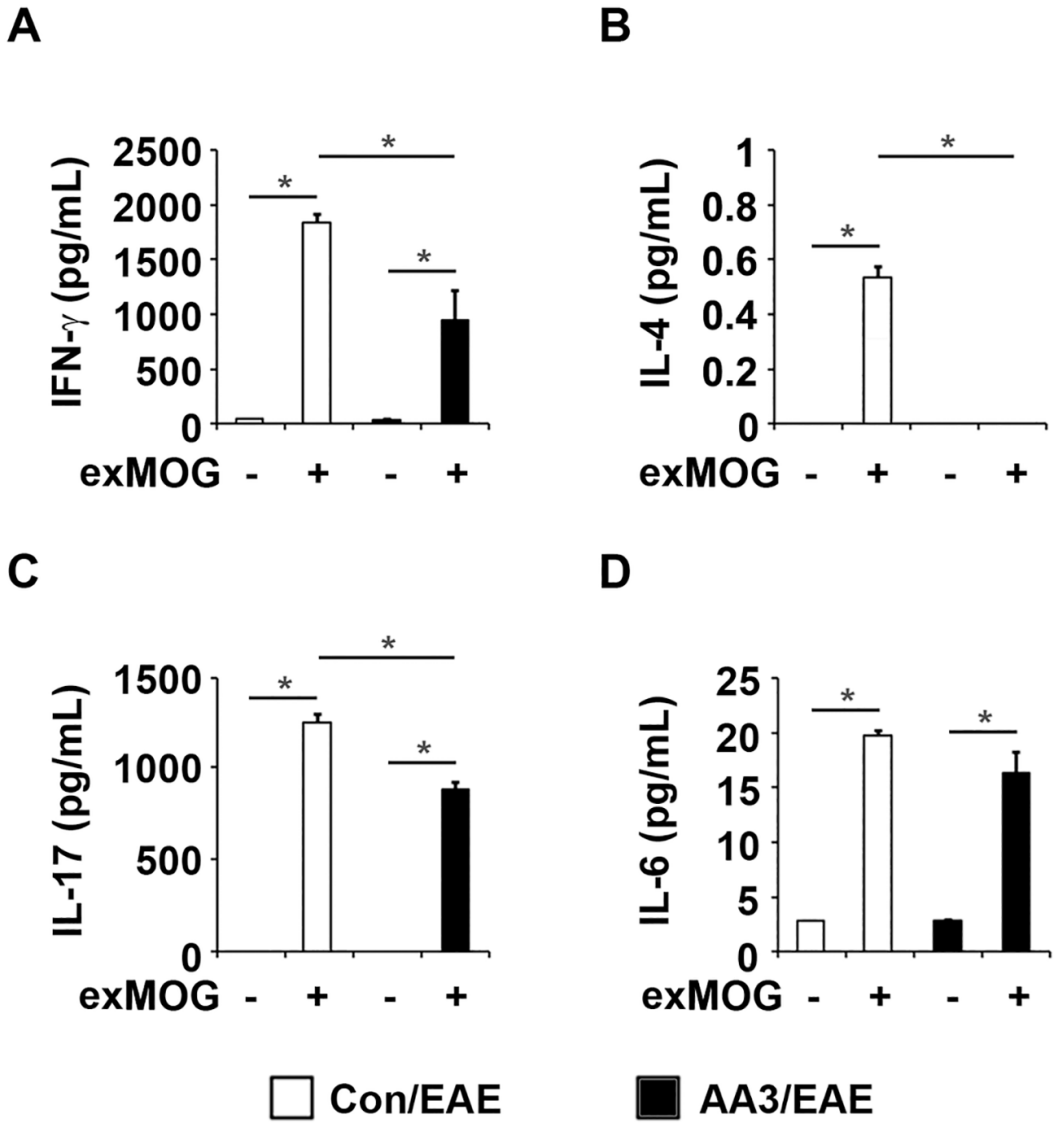
Oral administration of AA3 reduces the inflammatory cytokine response of lymphocytes upon MOG_35–55_ re-stimulation. We stimulated primary lymphocytes isolated from spleens of the control EAE mice (open bar) or the AA3-treated EAE mice (black bar) with MOG_35–55_ peptide (exMOG) for 2 days. We quantified cytokine expression in the conditioned media by ELISA; the expression of (A) IFN-γ, (B) IL-4, (C) IL-17, and (D) IL-6 are shown. Data are mean ± SEM (*n* = 24 per treatment group); *p < 0.05, one-way ANOVA.

Recent findings suggest that the predominance of Th1- and Th17-type immune responses play important roles in the pathogenesis of EAE [6, 7, 9, 27]. To examine the mechanistic actions of AA3 in alleviating EAE severity in mice, we examined the levels of the respective signature cytokines of the Th1, Th2, and Th17 pathways in cultured lymphocytes derived from the spleens of EAE mice. MOG_35–55_ re-challenge increased IFN-γ, IL-4, IL-17, and IL-6 levels in lymphocyte cultures from the control EAE mice (Fig 3A–3D). However, IFN-γ, IL-4, and IL-17 levels were reduced in lymphocyte cultures from the AA3-treated EAE mice after MOG_35–55_ re-stimulation (Fig 3A–3C). Although IL-6 level was also lower in cultures from the AA3-treated mice, the difference was not statistically significant (Fig 3D). These results collectively demonstrate that AA3 modulates the Th1, Th2, and Th17 cytokine responses in EAE mice.

**Fig 4:**
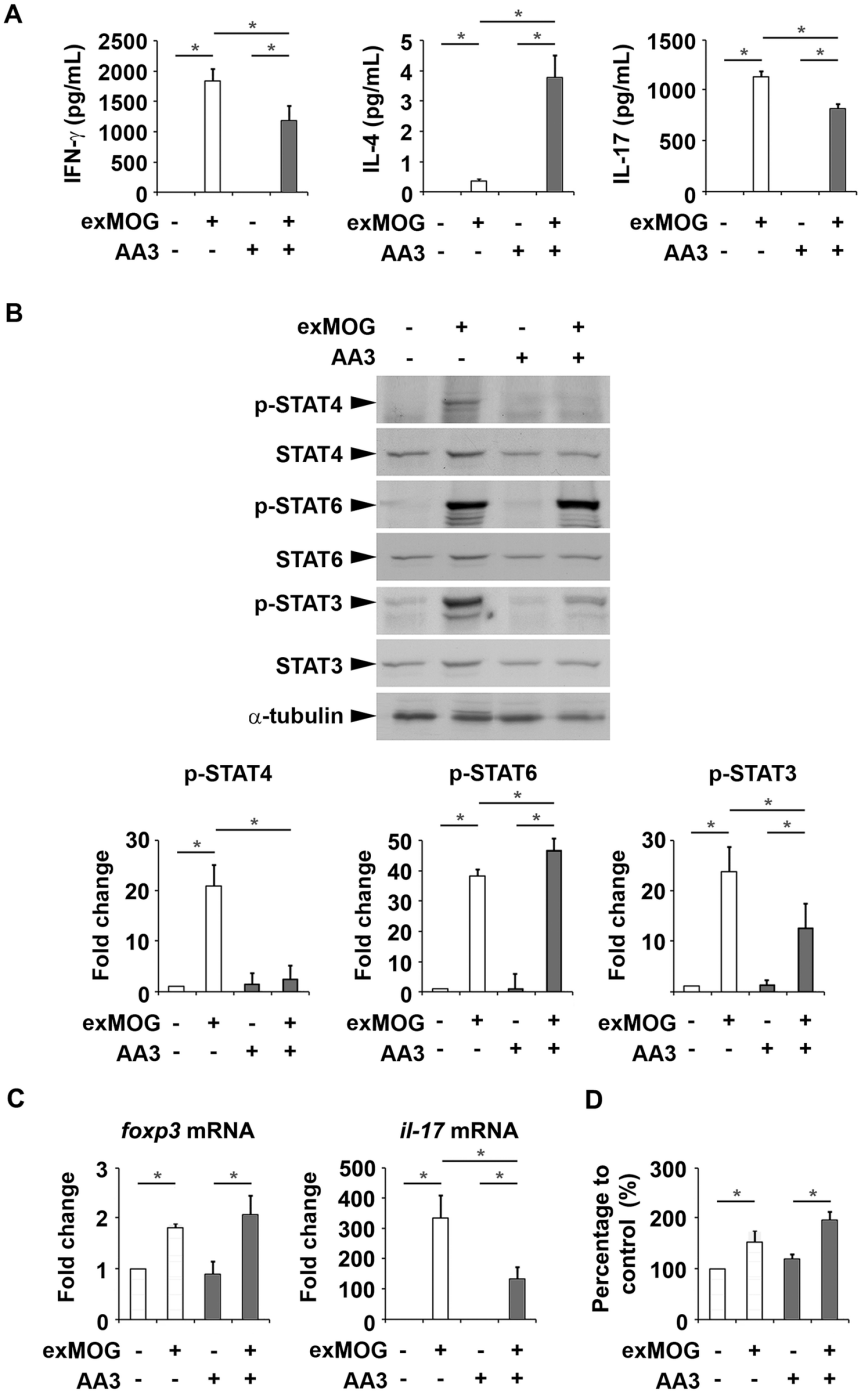
AA3 attenuates the production of pro-inflammatory cytokines in MOG-reactive lymphocytes. (A) We re-challenged primary lymphocytes from EAE mice with MOG_35–55_ peptide in the presence of AA3. We quantified IFN-γ, IL-4, and IL-17 levels in the culture supernatants by ELISA. (B) Western blots of phosphorylated and total STAT4, STAT6, and STAT3 from whole-cell lysates collected from the cultures in (A). α-tubulin served as an equal loading control. Densitometric analysis of signals from western blots in (B). (C) qPCR analysis of *foxp3* and *il-17* in the MOG-reactive lymphocytes. (D) We examined the cell survival of the MOG-reactive lymphocytes by MTT assay. Data are mean ± SEM (*n* = 3 per treatment group); *p < 0.05, one-way ANOVA.

We subsequently determined if AA3 directly affects the reaction of MOG-responsive lymphocytes (Fig 4A). The addition of MOG_35–55_ to the lymphocyte cultures induced IFN-γ release, which was significantly reduced in the presence of AA3. Similar to its effect on IFN-γ, MOG_35–55_ induced IL-17 release, which was downregulated by AA3. Interestingly, AA3 enhanced IL-4 induction in cultured lymphocytes from the control EAE mice.

Emerging evidence suggests that STAT family transcription factors play critical roles in T helper cell proliferation and differentiation. In particular, STAT4 is a key signaling molecule essential for Th1 cell differentiation [28]; STAT6 promotes Th2 cell differentiation [28–30], and the activation of STAT3 facilitates Th17 lineage commitment [31]. Accordingly, we examined if AA3 regulates the upstream STAT signaling in lymphocytes cultured from EAE mice (Fig 4B). MOG_35–55_ administration induced STAT4 and STAT3 phosphorylation in EAE lymphocytes, whereas the induction was greatly reduced in the presence of AA3. Consistent with the induction of IL-4, MOG_35–55_ treatment resulted in STAT6 phosphorylation, which was slightly potentiated by AA3.

The imbalance between regulatory T (Treg) and Th17 cells was recently identified as a critical factor for the development of EAE and human MS [32, 33]. Therefore, we examined if AA3 promotes the Treg cell response in EAE. We isolated total RNA from EAE lymphocytes after MOG_35–55_ treatment and subjected them to qPCR for the Treg cell marker gene, *foxp3* [23], and the Th17 cell marker gene, *il-17*. MOG_35–55_ treatment induced the expression of *foxp3* and *il-17* mRNA in EAE lymphocytes (Fig 4C). However, AA3 did not affect *foxp3* gene expression but significantly suppressed *il-17* gene expression. Thus, our results suggest that AA3 does not shift the balance between Treg and Th17 cells.

AA3 administration did not affect the overall cell survival of lymphocytes isolated from EAE mice (Fig 4D), suggesting that AA3 affects the MOG-reactive T cell responses but not proliferation. In summary, these results suggest an intriguing effect of AA3 on EAE wherein AA3 modulates Th1, Th2, and Th17 responses.

### AA3 inhibits Th17 cell differentiation

**Fig 5:**
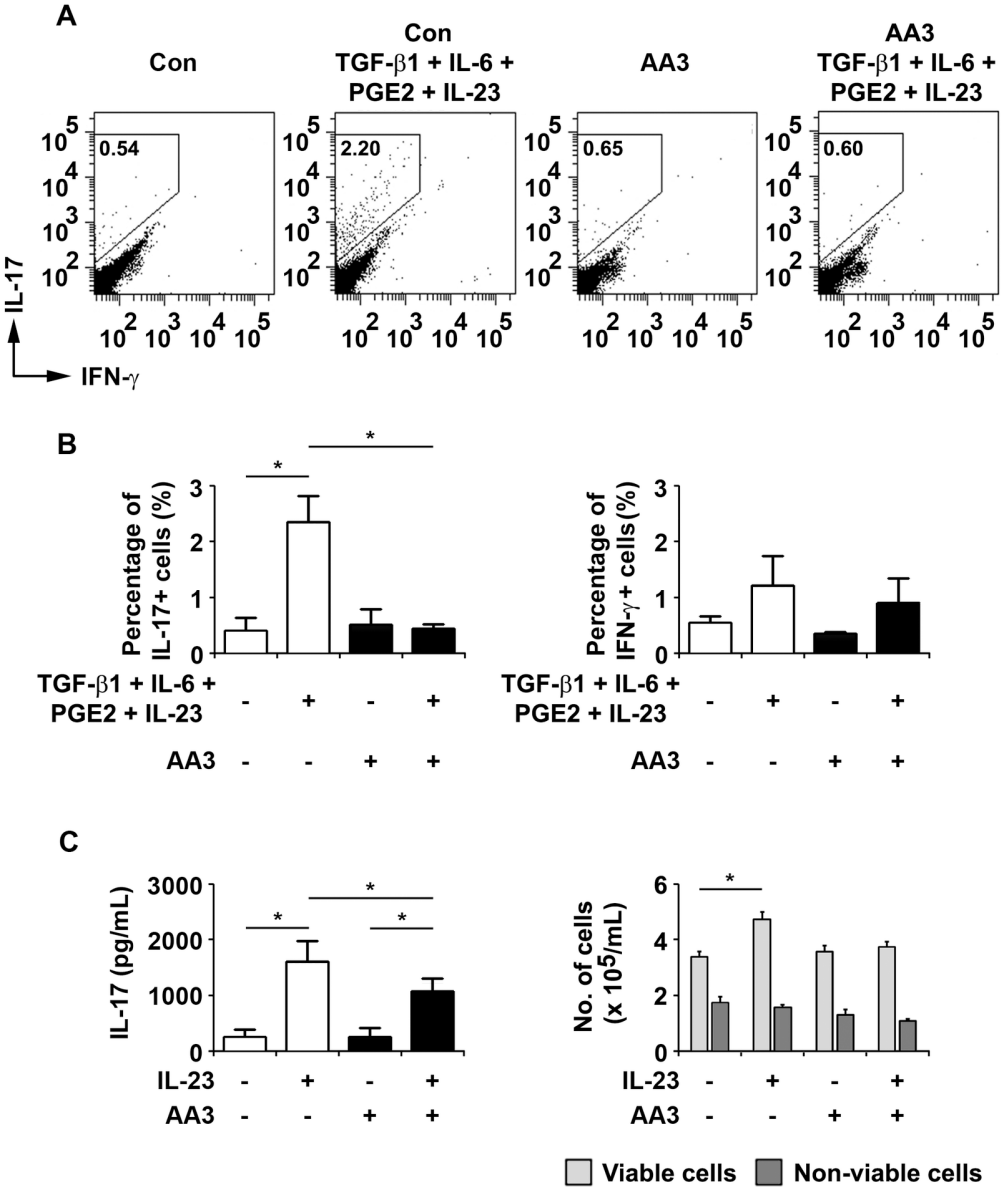
AA3 reduces Th17 cell differentiation. (A) We stimulated T helper cells isolated from naïve mice with TGF-β and IL-6, followed by IL-23 and PGE2, in the presence or absence of AA3 in order to differentiate them into Th17 cells. We analyzed IL-17–positive cells by flow cytometry after intracellular staining for IFN-γ and IL-17. Cells without AA3 treatment were used as a control. Representative cytograms are shown. (B) Histograms of the percentages of CD4+ IL-17^+^ IFN-γ^−^ and CD4^+^ IL-17^−^ IFN-γ^+^ cells obtained from the experiment conducted in (A). (C) We stimulated T helper cells with IL-23 to induce IL-17 cell expansion in the presence or absence of AA3. We quantified IL-17 levels in the culture media by ELISA and determined the numbers of viable and non-viable cells by Trypan Blue staining. Data are mean ± SEM (*n* = 3 per treatment group); *p < 0.05, one-way ANOVA.

TGF-β and IL-6 can induce the differentiation of Th17 cells, and IL-23 is necessary for the expansion and maintenance of Th17 cells [6, 34]. Furthermore, PGE2–EP4 activation can further amplify IL-23–mediated Th17 cell expansion, thereby promoting inflammation in EAE [7]. To elucidate the molecular mechanisms of AA3 in EAE, we examined the direct effect of AA3 on Th17 cell development. We treated naïve T cells differentiated by TGF-β and IL-6 with AA3 for 3 days, followed by the addition of IL-23, PGE2, and AA3 for another 3 days. Flow cytometric analysis of the percentage of IL-17^+^ and IFN-γ^−^ T cells showed that AA3 treatment greatly reduced the percentage of IL-17–producing cells upon cytokine stimulation (Fig 5A and 5B). Furthermore, ELISA revealed that AA3 significantly inhibited IL-23–mediated Th17 cell expansion as shown by the reduction of IL-17 secretion (Fig 5C). AA3 treatment did not affect the cell survival of CD4^+^ cells (Fig 5C), indicating that the reduction of Th17 population upon AA3 treatment might not be due to cell death. These results collectively suggest that AA3 attenuates the severity of EAE via the direct inhibition of Th17 cell differentiation.

## Discussion

The present study is the first to demonstrate that AA3 modulates the PGE2–EP4 signaling pathway. Moreover, by using the EAE model of MS, we found that AA3 administration reduces the clinical manifestations of EAE through the downregulation of inflammatory cytokines, particularly in Th1 and Th17 cells. Mechanistically, AA3 blocks PGE2-mediated Th17 cell differentiation and IL-23–induced Th17 expansion, which contribute to its beneficial effects on EAE.

There is no cure for MS, as available treatments merely relieve symptoms and retard disease progression. Specifically, current treatment strategies primarily focus on the inflammatory component of the disease [35, 36]; the actions of these drugs include anti-inflammatory effects (e.g., IFN-β-1a and glatiramer acetate), inhibition of pathogenic lymphocyte proliferation and expansion (e.g., teriflunomide), prevention of lymphocyte migration from the bloodstream to the CNS (e.g., fingolimod and natalizumab), and depletion of T and B lymphocytes (e.g., alemtuzumab and ocrelizumab). However, there is no approved treatment that reduces neuronal damage or promotes repair. We previously reported that AA3 acts as a non-competitive NMDA receptor modulator to protect neurons against ischemic brain injury and excitotoxicity in rats [21]. In addition to the anti-inflammatory effect of AA3 shown in the present study, the previously demonstrated neuroprotective and cognition-enhancing effects of AA3 [21] may provide additional benefit to patients with MS, because cognitive impairment is a common clinical feature in both the earlier and later phases of the disease [37]. Because of the complex nature of the pathogenesis of MS and neurodegeneration in general, multifunctional drugs with both neuroprotective and anti-inflammatory properties, such as AA3, are attracting significant attention in drug development for MS. New therapeutic strategies for MS, including immunological tolerance induction [38–40], cell-based therapy [41, 42], microbiota therapies [43], blood—brain barrier protection [44, 45], and demyelination prevention [46, 47] have recently been validated experimentally and clinically. Accordingly, it is of interest to determine if AA3 exhibits effects beyond the effects of the abovementioned novel treatment approaches for MS.

Our findings indicate that AA3 exerts an anti-inflammatory effect and ameliorates EAE severity. Th1/Th2 cytokine imbalance plays a critical role in the pathogeneses of EAE and MS, and much effort has been spared aiming to rebalance Th1/Th2 cellular responses to treat MS [48–50]. Moreover, both EAE and MS exhibit Th17-driven inflammatory responses, highlighting a potential target for the treatment of MS [7, 51]. In the present study, AA3 inhibited the inflammatory Th1 and Th17 responses as indicated by the reduction of IFN-γ and IL-17 released from lymphocytes isolated from the control EAE and AA3-treated EAE mice after MOG_35–55_ re-stimulation. Moreover, MOG_35–55_ re-stimulation and AA3 treatment induced IL-4 expression in lymphocytes from EAE mice, suggesting that there was a shift from a Th1 to a Th2 response after direct AA3 administration in MOG-responsive T cells. However, we did not observe IL-4 induction in lymphocytes isolated from the AA3-treated EAE mice. This might reflect the complexity of the regulation of IL-4 by AA3. The effect of AA3 on IL-4 might be transient, or a second negative regulator may exist *in vivo*. Nevertheless, the present results support the notion that restoring Th1/Th2 balance is important for the treatment of EAE and that the therapeutic effect of AA3 in EAE mice could drive the Th1/Th2 balance towards a Th2 anti-inflammatory response.

Our study also provides the first evidence that AA3 modulates the activities of PGE2 and EP4 signaling. In the EAE model, NSAIDs more effectively inhibit EAE pathology with selective COX-2 inhibitors than with mixed COX-1/2 inhibitors [52–55]. However, no clinical studies have demonstrated a beneficial effect of COX inhibitors for patients with MS [56]. NSAIDs are commonly prescribed to patients with MS to ameliorate the adverse flu-like effects associated with immunosuppressive drugs such as IFN-β [57, 58]. On the basis of our results, AA3 may be a good therapeutic candidate for MS. Unlike NSAIDs, which inhibit PGE2 synthesis, AA3 inhibits PGE2 signaling at the receptor level. AA3, at least in part, attenuates PGE2 function via the inhibition of EP4 signaling. AA3 also inhibits PGE2-promoted Th17 cell expansion. These results provide a rational basis for further testing the drug efficiency of AA3 for the treatment of MS.

In conclusion, AA3, a natural triterpenoid glycoside, has anti-inflammatory activity, particularly against EAE clinical pathology. From a wider perspective, the striking neuroprotective, cognitive-enhancing, and anti-inflammatory properties of AA3 suggest that it is a therapeutic candidate for neurodegenerative disorders besides MS. Thus, AA3 might be particularly useful for neuroprotection in other diseases of the central and peripheral nervous systems that are pathogenically characterized by inflammation and synaptic dysfunction. Accordingly, further studies on the underlying molecular mechanisms of AA3 will help optimize its therapeutic effect for MS.

## Abbreviations

AA3: anemoside A3
cAMP: cyclic adenosine monophosphate
COX: cyclooxygenase
EAE: experimental autoimmune encephalomyelitis
ELISA: enzyme-linked immunosorbent assay
EP: prostaglandin E2 receptor
*foxp3*: forkhead box P3
*gapdh*: glyceraldehyde-3-phosphate dehydrogenase
IC_50_: half maximal inhibitory concentration
IFN: interferon
IL: interleukin
MOG: myelin oligodendrocyte glycoprotein
MS: multiple sclerosis
NMDA: *N*-methyl-d-aspartate
PGE2: prostaglandin E2
STAT: signal transducer and activator of transcription
Th: T helper
TNF: tumor necrosis factor
Treg: regulatory T

## References

[1] Hemmer B, Archelos JJ, Hartung HP. New concepts in the immunopathogenesis of multiple sclerosis. Nat Rev Neurosci. 2002;3: 291–301. doi: 10.1038/nrn784 11967559 10.1038/nrn784

[2] Degenhardt A, Ramagopalan SV, Scalfari A, Ebers GC. Clinical prognostic factors in multiple sclerosis: a natural history review. Nat Rev Neurol. 2009;5: 672–682. doi: 10.1038/nrneurol.2009.178 19953117 10.1038/nrneurol.2009.178

[3] Mahad DH, Trapp BD, Lassmann H. Pathological mechanisms in progressive multiple sclerosis. Lancet Neurol. 2015;14: 183–193. doi: 10.1016/S1474-4422(14)70256-X 25772897 10.1016/S1474-4422(14)70256-X

[4] Popescu BF, Pirko I, Lucchinetti CF. Pathology of multiple sclerosis: where do we stand? Continuum (Minneap Minn). 2013;19: 901–921. 23917093 10.1212/01.CON.0000433291.23091.65 PMC3915566

[5] Franklin RJ, ffrench-Constant C, Edgar JM, Smith KJ. Neuroprotection and repair in multiple sclerosis. Nat Rev Neurol. 2012;8: 624–634. doi: 10.1038/nrneurol.2012.200 23026979 10.1038/nrneurol.2012.200

[6] Steinman L. A brief history of T(H)17, the first major revision in the T(H)1/T(H)2 hypothesis of T cell-mediated tissue damage. Nat Med. 2007;13: 139–145. doi: 10.1038/nm1551 17290272 10.1038/nm1551

[7] Yao C, Sakata D, Esaki Y, Li Y, Matsuoka T, Kuroiwa K, et al Prostaglandin E2-EP4 signaling promotes immune inflammation through Th1 cell differentiation and Th17 cell expansion. Nat Med. 2009;15: 633–640. doi: 10.1038/nm.1968 19465928 10.1038/nm.1968

[8] Fletcher JM, Lalor SJ, Sweeney CM, Tubridy N, Mills KH. T cells in multiple sclerosis and experimental autoimmune encephalomyelitis. Clin Exp Immunol. 2010;162: 1–11. doi: 10.1111/j.1365-2249.2010.04143.x 20682002 10.1111/j.1365-2249.2010.04143.x PMC2990924

[9] Lovett-Racke AE, Yang Y, Racke MK. Th1 versus Th17: are T cell cytokines relevant in multiple sclerosis? Biochim Biophys Acta. 2011;1812: 246–251. doi: 10.1016/j.bbadis.2010.05.012 20600875 10.1016/j.bbadis.2010.05.012 PMC3004998

[10] Kihara Y, Matsushita T, Kita Y, Uematsu S, Akira S, Kira J, et al Targeted lipidomics reveals mPGES-1-PGE2 as a therapeutic target for multiple sclerosis. Proc Natl Acad Sci U S A. 2009;106: 21807–21812. doi: 10.1073/pnas.0906891106 19995978 10.1073/pnas.0906891106 PMC2789753

[11] Esaki Y, Li Y, Sakata D, Yao C, Segi-Nishida E, Matsuoka T, et al Dual roles of PGE2-EP4 signaling in mouse experimental autoimmune encephalomyelitis. Proc Natl Acad Sci U S A. 2010;107: 12233–12238. doi: 10.1073/pnas.0915112107 20566843 10.1073/pnas.0915112107 PMC2901475

[12] Cudaback E, Jorstad NL, Yang Y, Montine TJ, Keene CD. Therapeutic implications of the prostaglandin pathway in Alzheimer's disease. Biochem Pharmacol. 2014;88: 565–572. doi: 10.1016/j.bcp.2013.12.014 24434190 10.1016/j.bcp.2013.12.014 PMC3972296

[13] Kawahara K, Hohjoh H, Inazumi T, Tsuchiya S, Sugimoto Y. Prostaglandin E2-induced inflammation: Relevance of prostaglandin E receptors. Biochim Biophys Acta. 2015;1851: 414–421. doi: 10.1016/j.bbalip.2014.07.008 25038274 10.1016/j.bbalip.2014.07.008

[14] Ye W., Ji NN, Zhao SX, Liu JH, Ye T, McKervey MA, et al Stevenson P. Triterpenoids from Pulsatilla chinensis. Phytochemistry 1996;42: 799–802. 8768325 10.1016/0031-9422(96)00043-x

[15] Zheng Y, Zhou F, Wu X, Wen X, Li Y, Yan B, et al 23-Hydroxybetulinic acid from Pulsatilla chinensis (Bunge) Regel synergizes the antitumor activities of doxorubicin in vitro and in vivo. J Ethnopharmacol. 2010;128: 615–622. doi: 10.1016/j.jep.2010.02.004 20176097 10.1016/j.jep.2010.02.004

[16] Cheng L, Zhang M, Zhang P, Song Z, Ma Z, Qu H. Silver complexation and tandem mass spectrometry for differentiation of triterpenoid saponins from the roots of Pulsatilla chinensis (Bunge) Regel. Rapid Commun Mass Spectrom. 2008;22: 3783–3790. doi: 10.1002/rcm.3801 18973198 10.1002/rcm.3801

[17] Xu H, Shi X, Ji X, Du Y, Zhu H, Zhang L. Qualitative and quantitative determination of nine main active constituents in Pulsatilla cernua by high-performance liquid chromatography coupled to electrospray ionization tandem mass spectrometry. J Sep Sci. 2011a;34: 308–316. 21268254 10.1002/jssc.201000660

[18] Xu H, Ji X, Shi X, Du Y, Zhu H, Zhang L. Development of a novel method for triterpenoidal saponins in rat plasma by solid-phase extraction and high-performance liquid chromatography tandem mass spectrometry. Anal Biochem. 2011b;419: 323–332. 21925476 10.1016/j.ab.2011.08.045

[19] Zhang DM, Lin SM, Lau CW, Yiu A, Wang J, Li Y, et al Anemoside A3-induced relaxation in rat renal arteries: role of endothelium and Ca2+ channel inhibition. Planta Med. 2010;76: 1814–1819. doi: 10.1055/s-0030-1250003 20506075 10.1055/s-0030-1250003

[20] Gao XD, Ye WC, Yu AC, Zhang Y, Tan RX, Li M, et al Pulsatilloside A and anemoside A3 protect PC12 cells from apoptosis induced by sodium cyanide and glucose deprivation. Planta Med. 2003;69: 171–174. doi: 10.1055/s-2003-37705 12624827 10.1055/s-2003-37705

[21] Ip FC, Fu WY, Cheng EY, Tong EP, Lok KC, Liang Y, et al Anemoside A3 Enhances Cognition through the Regulation of Synaptic Function and Neuroprotection. Neuropsychopharmacology. 2015;40: 1877–1887. doi: 10.1038/npp.2015.37 25649278 10.1038/npp.2015.37 PMC4839511

[22] Stromnes IM, Goverman JM. Active induction of experimental allergic encephalomyelitis. Nat Protoc. 2006;1: 1810–1819. doi: 10.1038/nprot.2006.285 17487163 10.1038/nprot.2006.285

[23] Ano S, Morishima Y, Ishii Y, Yoh K, Yageta Y, Ohtsuka S, et al Transcription factors GATA-3 and RORγt are important for determining the phenotype of allergic airway inflammation in a murine model of asthma. J Immunol. 2013;190: 1056–1065. doi: 10.4049/jimmunol.1202386 23293351 10.4049/jimmunol.1202386

[24] Nagashima H, Okuyama Y, Asao A, Kawabe T, Yamaki S, Nakano H, et al The adaptor TRAF5 limits the differentiation of inflammatory CD4(+) T cells by antagonizing signaling via the receptor for IL-6. Nat Immunol. 2014;15: 449–456. doi: 10.1038/ni.2863 24681564 10.1038/ni.2863 PMC4108451

[25] Kipp M, van der Star B, Vogel DY, Puentes F, van der Valk P, Baker, Amor S. Experimental in vivo and in vitro models of multiple sclerosis: EAE and beyond. Mult Scler Relat Disord. 2012;1: 15–28. doi: 10.1016/j.msard.2011.09.002 25876447 10.1016/j.msard.2011.09.002

[26] Baker D, Amor S. Mouse models of multiple sclerosis: lost in translation? Curr Pharm Des. 2015;21: 2440–2452. 25777759 10.2174/1381612821666150316122706

[27] El-Behi M, Rostami A, Ciric B. Current Views on the Roles of Th1 and Th17 Cells in Experimental Autoimmune Encephalomyelitis. J Neuroimmune Pharmacol. 2010;5: 189–197. doi: 10.1007/s11481-009-9188-9 20107924 10.1007/s11481-009-9188-9 PMC2866798

[28] Chitnis T, Najafian N, Benou C, Salama AD, Grusb MJ, Sayegh MH, et al Effect of targeted disruption of STAT4 and STAT6 on the induction of experimental autoimmune encephalomyelitis. J Clin Invest. 2001;108: 739–747. doi: 10.1172/JCI12563 11544280 10.1172/JCI12563 PMC209380

[29] Forbes E, van Panhuys N, Min B, Le Gros G. Differential requirements for IL-4/STAT6 signalling in CD4 T-cell fate determination and Th2-immune effector responses. Immunol Cell Biol. 2010;88: 240–243. doi: 10.1038/icb.2009.101 20010912 10.1038/icb.2009.101

[30] Maier E, Duschl A, Horejs-Hoeck J. STAT6-dependent and -independent mechanisms in Th2 polarization. Eur J Immunol. 2012;42: 2827–2833. doi: 10.1002/eji.201242433 23041833 10.1002/eji.201242433 PMC3557721

[31] Hirahara K, Ghoreschi K, Laurence A, Yang XP, Kanno Y, O’Shea JJ. Signal transduction pathways and transcriptional regulation in Th17 cell differentiation. Cytokine Growth Factor Rev. 2010;21: 425–434. doi: 10.1016/j.cytogfr.2010.10.006 21084214 10.1016/j.cytogfr.2010.10.006 PMC3182452

[32] Fletcher JM, Lonergan R, Costelloe L, Kinsella K, Moran B, O’Farrelly C, et al CD39+Foxp3+ regulatory T Cells suppress pathogenic Th17 cells and are impaired in multiple sclerosis. J Immunol. 2009;183: 7602–7610. doi: 10.4049/jimmunol.0901881 19917691 10.4049/jimmunol.0901881

[33] Ulges A, Witsch EJ, Pramanik G, Klein M, Birkner K, Bühler U, et al Protein kinase CK2 governs the molecular decision between encephalitogenic TH17 cell and Treg cell development. Proc Natl Acad Sci U S A. 2016;113: 10145–10150. doi: 10.1073/pnas.1523869113 27555590 10.1073/pnas.1523869113 PMC5018788

[34] Morishima N, Mizoguchi I, Takeda K, Mizuguchi J, Yoshimoto T. TGF-beta is necessary for induction of IL-23R and Th17 differentiation by IL-6 and IL-23. Biochem Biophys Res Commun. 2009;386: 105–110. doi: 10.1016/j.bbrc.2009.05.140 19501566 10.1016/j.bbrc.2009.05.140

[35] Bridel C, Lalive PH. Update on multiple sclerosis treatments. Swiss Med Wkly. 2014;144: w14012 doi: 10.4414/smw.2014.14012 25247669 10.4414/smw.2014.14012

[36] English C, Aloi JJ. New FDA-Approved Disease-Modifying Therapies for Multiple Sclerosis. Clin Ther. 2015;37: 691–715. doi: 10.1016/j.clinthera.2015.03.001 25846320 10.1016/j.clinthera.2015.03.001

[37] DeLuca GC, Yates RL, Beale H, Morrow SA. Cognitive impairment in multiple sclerosis: clinical, radiologic and pathologic insights. Brain Pathol. 2015;25: 79–98. doi: 10.1111/bpa.12220 25521179 10.1111/bpa.12220 PMC8029470

[38] Kohm AP, Williams JS, Bickford AL, McMahon JS, Chatenoud L, Bach JF, et al Treatment with nonmitogenic anti-CD3 monoclonal antibody induces CD4+ T cell unresponsiveness and functional reversal of established experimental autoimmune encephalomyelitis. J Immunol. 2005;174: 4525–4534. 15814673 10.4049/jimmunol.174.8.4525

[39] Farooq SM, Ashour HM. Eye-mediated induction of specific immune tolerance to encephalitogenic antigens. CNS Neurosci Ther. 2013;19: 503–510. doi: 10.1111/cns.12087 23522052 10.1111/cns.12087 PMC6493606

[40] Anderton SM. Peptide immunotherapy in experimental autoimmune encephalomyelitis. Biomed J. 2015;38: 206–214. doi: 10.4103/2319-4170.158510 26068029 10.4103/2319-4170.158510

[41] Gharibi T, Ahmadi M, Seyfizadeh N, Jadidi-Niaragh F, Yousefi M. Immunomodulatory characteristics of mesenchymal stem cells and their role in the treatment of multiple sclerosis. Cell Immunol. 2015;293: 113–121. doi: 10.1016/j.cellimm.2015.01.002 25596473 10.1016/j.cellimm.2015.01.002

[42] Cheng Y, Sun L, Xie Z, Fan X, Cao Q, Han J, et al Diversity of immune cell types in multiple sclerosis and its animal model: Pathological and therapeutic implications. J Neurosci Res. 2017 doi: 10.1002/jnr.24023 [Epub ahead of print] 28084640 10.1002/jnr.24023 PMC5573979

[43] Mirza A, Mao-Draayer Y. The gut microbiome and microbial translocation in multiple sclerosis. Clin Immunol. 2017 3 9 doi: 10.1016/j.clim.2017.03.001 [Epub ahead of print] 28286112 10.1016/j.clim.2017.03.001

[44] Alvarez JI, Cayrol R, Prat A. Disruption of central nervous system barriers in multiple sclerosis. Biochim Biophys Acta. 2011;1812: 252–264. doi: 10.1016/j.bbadis.2010.06.017 20619340 10.1016/j.bbadis.2010.06.017

[45] Lopes Pinheiro MA, Kooij G, Mizee MR, Kamermans A, Enzmann G, Lyck R, et al Immune cell trafficking across the barriers of the central nervous system in multiple sclerosis and stroke. Biochim Biophys Acta. 2016;1862: 461–471. doi: 10.1016/j.bbadis.2015.10.018 26527183 10.1016/j.bbadis.2015.10.018

[46] Olsen JA, Akirav EM. Remyelination in multiple sclerosis: cellular mechanisms and novel therapeutic approaches. J Neurosci Res. 2015;93: 687–696. doi: 10.1002/jnr.23493 25287108 10.1002/jnr.23493

[47] Chamberlain KA, Nanescu SE, Psachoulia K, Huang JK. Oligodendrocyte regeneration: Its significance in myelin replacement and neuroprotection in multiple sclerosis. Neuropharmacology. 2016;110: 633–643. doi: 10.1016/j.neuropharm.2015.10.010 26474658 10.1016/j.neuropharm.2015.10.010 PMC4841742

[48] Butti E, Bergami A, Recchia A, Brambilla E, Del Carro U, Amadio S, et al IL4 gene delivery to the CNS recruits regulatory T cells and induces clinical recovery in mouse models of multiple sclerosis. Gene Ther. 2008;15: 504–515. doi: 10.1038/gt.2008.10 18239607 10.1038/gt.2008.10

[49] Rostami A, Ciric B. Role of Th17 cells in the pathogenesis of CNS inflammatory demyelination. Neurol Sci. 2013;333: 76–87. 10.1016/j.jns.2013.03.002 PMC3726569 23578791

[50] Avsar T, Durası İM, Uygunoğlu U, Tütüncü M, Demirci NO, Saip S, et al CSF Proteomics Identifies Specific and Shared Pathways for Multiple Sclerosis Clinical Subtypes. PLoS One. 2015;10: e0122045 doi: 10.1371/journal.pone.0122045 25942430 10.1371/journal.pone.0122045 PMC4420287

[51] Yadav SK, Mindur JE, Ito K, Dhib-Jalbut S. Advances in the immunopathogenesis of multiple sclerosis. Curr Opin Neurol. 2015;28: 206–219. doi: 10.1097/WCO.0000000000000205 25887768 10.1097/WCO.0000000000000205

[52] Miyamoto K, Miyake S, Mizuno M, Oka N, Kusunoki S, Yamamura T. Selective COX-2 inhibitor celecoxib prevents experimental autoimmune encephalomyelitis through COX-2-independent pathway. Brain. 2006;129: 1984–1992. doi: 10.1093/brain/awl170 16835249 10.1093/brain/awl170

[53] Muthian G, Raikwar HP, Johnson C, Rajasingh J, Kalgutkar A, Marnett LJ, et al COX-2 inhibitors modulate IL-12 signaling through JAK-STAT pathway leading to Th1 response in experimental allergic encephalomyelitis. J. Clin. Immunol. 2006;26: 73–85. doi: 10.1007/s10875-006-8787-y 16418805 10.1007/s10875-006-8787-y

[54] Ni J, Shu YY, Zhu YN, Fu YF, Tang W, Zhong XG, et al COX-2 inhibitors ameliorate experimental autoimmune encephalomyelitis through modulating IFN-gamma and IL-10 production by inhibiting T-bet expression. J. Neuroimmunol. 2007;186: 94–103. doi: 10.1016/j.jneuroim.2007.03.012 17442406 10.1016/j.jneuroim.2007.03.012

[55] Palumbo S, Bosetti F. Alterations of brain eicosanoid synthetic pathway in multiple sclerosis and in animal models of demyelination: role of cyclooxygenase-2. Prostaglandins Leukot Essent Fatty Acids. 2013;89: 273–278. doi: 10.1016/j.plefa.2013.08.008 24095587 10.1016/j.plefa.2013.08.008

[56] Mora JS, Kao KP, Munsat TL. Indomethacin reduces the side effects of intrathecal interferon. N. Engl. J. Med. 1984;310: 126–127. doi: 10.1056/NEJM198401123100219 6197650 10.1056/NEJM198401123100219

[57] Reess J, Haas J, Gabriel K, Fuhlrott A, Fiola M. Both paracetamol and ibuprofen are equally effective in managing flu-like symptoms in relapsing-remitting multiple sclerosis patients during interferon beta-1a (AVONEX) therapy. Mult Scler. 2002;8: 15–18. doi: 10.1191/1352458502ms771sr 11936482 10.1191/1352458502ms771sr

[58] Río J, Nos C, Bonaventura I, Arroyo R, Genis D, Sureda B, et al Corticosteroids, ibuprofen, and acetaminophen for IFNbeta-1a flu symptoms in MS: a randomized trial. Neurology. 2004;63: 525–528. 15304586 10.1212/01.wnl.0000133206.44931.25

